# Frequency of Non-Type I/II *CALR* Mutations in Patients Undergoing Molecular Diagnostics for Myeloproliferative Neoplasms

**DOI:** 10.3390/diseases14060190

**Published:** 2026-05-28

**Authors:** Tatiana Makarik, Elena Nikulina, Svetlana Treglasova, Elena Stepanova, Natalia Chernova, Bella Biderman, Alina Kokhno, Andrey Sudarikov

**Affiliations:** 1 National Medical Research Center for Hematology, 125167 Moscow, Russia; makarik_71@mail.ru (T.M.); lenysh2007@rambler.ru (E.N.); svetik997@yandex.ru (S.T.); stepanova173@gmail.com (E.S.); bella_biderman@mail.ru (B.B.); anilako@rambler.ru (A.K.); 2 Moscow Multidisciplinary Clinical Center “Kommunarka”, 108814 Moscow, Russia; ngchernova@mail.ru

**Keywords:** calreticulin (CALR), myeloproliferative neoplasms (MPN)

## Abstract

Background/Objectives: Somatic *CALR* gene insertions/deletions in exon 9, causing frameshift, are a diagnostic sign of myeloproliferative neoplasms (MPNs). Besides the most common somatic mutations of type I (52 bp deletion) and type II (5 bp insertion), there are rare ones whose significance is not always clear. This study evaluates the frequency of rare mutations and demonstrates a germline rather than somatic nature for some of them. Methods: A retrospective analysis of 8417 blood samples subjected to molecular diagnosis of myeloproliferative neoplasm (MPN) was performed. Cases suspected as germline variants were sequenced, and paired samples (when available) of buccal epithelium were analyzed. Results: We have identified 632 C*ALR* gene mutation-positive cases. Most of the cases were typical insertions/deletions (5 bp/52 bp). Non-type I/II frameshift or nonframeshift mutations were observed in 68 cases (11%). The buccal swab samples obtained from 4 patients confirmed the germline nature of these variants. It is worth noting that the MPN diagnosis for three of these patients was made considering the presence of the *JAK2* V617F mutation (two cases) or *BCR::ABL1* translocation (one case). In one case, the diagnosis of MPN was reclassified to CML. Conclusions: Non-type I/II *CALR* mutations, according to our data, could be found in 0.8% of MPN-suspected cases, and may not be associated with the diagnosis. The detection of a non-standard *CALR* mutation with an allelic frequency close to 50% should raise suspicion of the possibility of a germline *CALR* variant, and such cases should be investigated further.

## 1. Introduction

Calreticulin is a calcium-binding chaperone that performs several functions in the immune response. In the endoplasmic reticulum, calreticulin promotes the folding of molecules of the main histocompatibility complex class I and their assembly factor tapazine, thereby affecting the presentation of antigens to cytotoxic T cells [1,2]. In 2013, the discovery of somatic mutations in the *CALR* gene, encoding calreticulin, in 70–84% of *JAK2* gene mutation-negative patients with essential thrombocythemia (ET) and primary myelofibrosis (PMF) significantly improved the molecular diagnosis of these diseases [3,4]. More than 50 somatic *CALR* mutations are known, which are insertions/deletions in exon 9 causing frameshift, which leads to the loss of the KDEL terminal signal [5]. The two most common mutations are type I, a 52 bp deletion (p. L367fs*46), and type II, a 5 bp TTGTC insertion (p. K385fs*47). The number of negatively charged amino acids in the C-terminal region of mutant proteins differs from that in wild-type calreticulin, depending on the mutation type. With a 5 bp insertion (type II), roughly half of the negatively charged amino acids are preserved, whereas 52 bp deletions (type I) eliminate nearly all such amino acids [3]. When mutant CALR interacts with the thrombopoietin MPL receptor, uncontrolled activation of the underlying JAK-STAT pathway and cellular transformation occur [6,7]. The value of *CALR* gene mutations as ET/PMF diagnostic criteria is defined by the WHO classification of myeloid neoplasms and acute leukemia in 2016 and 2022 [8,9,10]. Type I mutations are more commonly found in PMF. Type II is more commonly associated with ET. Type I mutations are associated with a more favorable prognosis, longer overall survival, and a lower risk of leukemic transformation compared to type II or *JAK2*-mutated PMF [11,12,13].

*CALR* gene mutations can be identified using several methods: capillary electrophoresis (also known as fragment analysis), real-time PCR, and next-generation sequencing (NGS). Both fragment analysis and NGS have uncovered numerous insertions and deletions in the *CALR* gene that went undetected with real-time PCR [14]. Earlier studies reported a low frequency of in-frame deletions and insertions in the *CALR* gene, which are assumed to preserve the terminal KDEL region of the CALR protein [15,16]. These in-frame variants often have an allelic frequency close to 50%, which may indicate a germline polymorphism. The importance of such variants for diagnosing MPNs is not yet clearly established.

A considerable number of publications discuss the investigation of *CALR* mutations in patient groups with a negative *JAK2* V617F status and suspected MPNs. In this study, most Phi-negative patients suspected of having MPN were tested for *JAK2* V617F, *CALR*, and *MPL* W515L/K mutations simultaneously. Furthermore, subsequently, we were able to find low *BCR::ABL* p210 expression in two patients with non-type I/II *CALR* gene deletion. Here, we outline the incidence of non-type I/II mutations in the *CALR* gene identified during diagnostic examinations of cases suspected to have classical MPN.

## 2. Materials and Methods

Somatic *CALR* gene mutations were evaluated for 8417 cases subjected to molecular diagnosis of MPN at the National Medical Research Center for Hematology (Moscow, Russia) from 2016 to 2026 and in a control group of 366 individuals without hematological diagnosis. All included cases were also tested for *JAK2* V616F, *MPL* W515L/K and *BCR::ABL1* chimeric transcript, essentially as described previously [17]. DNA and RNA were isolated from 5 to 10 mL of blood and bone marrow using standard salt extraction [18] and using a set of Ribosol-D reagents (Interlabservice, Moscow, Russia). DNA from buccal epithelium was isolated by direct cell lysis with proteinase K, followed by purification with magnetic particles (Pure blood DNA, Syntol, Moscow, Russia) in accordance with the manufacturer’s protocol. The mutation status of exon 9 of the *CALR* gene was evaluated using fragment length analysis after PCR with fluorescent primers in accordance with the method described by Klampfl et al. [3]. For the amplification of the target *CALR* gene sequence, primers *CALR*_AF1 5′-CTGAGGTGTGTGCTCTGCC-3′ and *CALR*_R FAM 5′-CAGAGACATTATTTGGCGCGG-3′ were used. DNA (200–600 ng) was added to 25 μL of reaction mixture (Syntol, Moscow, Russia), containing: 10 pmol of primers, H_2_O 14.5 μL, MgCl_2_ 25 mM–1.5 μL, dNTP Mix, concentration of each nucleotide 25 mM–2.5 μL, PCR Buffer-B for Taq DNA polymerase–2.5 μL, Taq DNA polymerase 0.25 μL per reaction. Amplification was performed in a C1000 thermocycler (Bio-Rad, Hercules, CA, USA): denaturation at 95 °C for 10 min, 35 cycles (95 °C for 20 s, 60 °C for 40 s, 72 °C for 60 s), 72 °C for 10 min. After amplification, the PCR mixture was diluted 100 times, denatured at 98 °C for 3 min, and cooled at 4 °C for 5 min. Subsequently, fragment analysis was performed using a Nanofor-05 genetic analyzer (Institute of Analytical Instrumentation of the Russian Academy of Sciences, St. Petersburg, Russia). The profile of the obtained amplicons (length distribution) and the degree of fluorescence were analyzed using GeneMapper v.4 software (Applied Biosystems, Waltham, MA, USA). The allele load (VAF%) was calculated based on the height of the mutant and wild-type peaks, determining the proportion of mutant alleles relative to the total number of alleles (mutant + wild-type) in the sample. Precise evaluation of the non-type I/II *CALR* gene insertions/deletions was done by targeted NGS. For the amplification of the target *CALR* gene sequence, the same primers and PCR conditions as for fragment analysis were used. NGS libraries were prepared using the “ShotGun Plus Kit ‘SG GM Plus’” (Sesana, Moscow, Russia). Sequencing was performed on a MiSeq genetic analyzer (Illumina, San Diego, CA, USA), bioinformatic analysis was performed using the open source software Trimmomatic (Version: 0.39), BWA (Version: 0.7.17-r1188), SAMtools (Version: 1.10 using htslib 1.20-30-g61b922b4), Vardict (Version: 1.8.2) and Annovar (Version: Date: 2020-06-07) [19,20,21,22,23]. The potential pathogenicity of the obtained variants was analyzed using the online databases Franklin by Genoox and SESHAT [24,25]. The *JAK2* V617F mutation was quantified by real-time allele-specific PCR using the “Kit of reagents for detecting the V617F G/T mutation of the *JAK2* gene” (Syntol, Russia) in accordance with the manufacturer’s instructions, with 0.2% detection sensitivity declared. Additionally, DNA from the UKE1 cell line was used as a positive control. *MPL* W515L/K mutations were evaluated by qualitative allele-specific real-time PCR using a “Set of reagents for determining the W515L/K mutation of the *MPL* gene” (Syntol, Moscow, Russia).

## 3. Results

A total of 8417 patients suspected of having MPN and 366 patients unrelated to MPN (control group) were tested for the presence of CALR gene variants. Demographic parameters of the patient sample are presented in Table 1.

**Figure 1:**
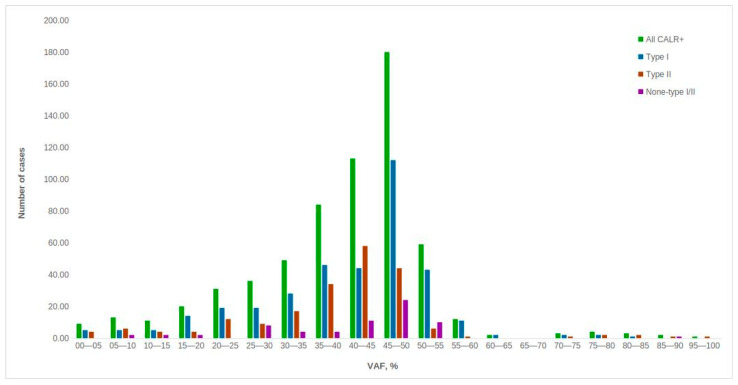
VAF for different types of *CALR* gene mutations.

As a control group, we analyzed 366 samples of non-hematological patients tested for thrombophilia markers (described previously elsewhere [26]). No *CALR* gene variants (including germline) were found in this group. The distribution of *CALR* mutations by type and VAF for 632 positive patients is shown in Figure 1. Mutations of type I accounted for 57% (360), type II—33% (209), and non-type I/II ones—10% (63). It should be noted that most of the mutations show an allelic load of 40–55%. Our findings are roughly consistent with those reported previously [9,27].

The frequency of *CALR* type I mutations was higher than that of *CALR* type II mutations, which aligns with the previously published data [13]. However, the number of detected non-type I/II *CALR* gene mutations in our patient sample was higher than what had been previously reported [5].

Nine cases with non-type I/II *CALR* gene mutations of exon 9 were further analyzed. Five were in-frame deletions. In four cases, frameshift mutations, including deletions of 13 bp and 31 bp, were observed. Because the study was retrospective, we were restricted in our ability to collect samples of non-hematopoietic tissue for cases involving *CALR* gene mutations that might be considered germline. Only for four of these cases were we able to obtain buccal swab samples. In all these cases, the same variants were found in the cells of the oral mucosa and peripheral blood with the same VAFs, which confirms the germline nature of these lesions. Such cases can certainly be considered as “triple-negative” and subjected to additional *JAK2* exon 12 sequencing. Clinical characteristics of patients with non-type I/II *CALR* mutations are presented in Table 2. The median VAF was 50% (range 45–52%). In six cases, an additional *JAK2* V617F mutation was found, and two cases were *BCR::ABL1* (p210) positive. It should be noted that in all these cases, the pathogenesis is linked specifically to additional mutations rather than *CALR* gene mutations. In the last two cases, the diagnosis of MPN was reclassified to chronic myeloid leukemia (CML).

All mutations were searched for in the Catalog of Somatic Mutations in Cancer (COSMIC) database and are labeled with the appropriate identity number if it existed at the time of search. The exact nomenclature for non-standard *CALR* mutations found in our patient cohort is presented in Table 3. Six mutations that were not found in the COSMIC database, to our knowledge, are newly identified mutations and therefore being reported for the first time. All four cases with buccal swab material available for evaluation appeared to be in-frame deletions.

As an example, we would like to give a description of a clinical case (patient No. 6 in Table 2 and Table 3). A 77-year-old patient with a diagnosis of MPN/MDS refractory anemia with excess blasts (RAEB-2) with pleural involvement was observed at the Moscow Multidisciplinary Clinical Center “Kommunarka”. Immunophenotype of blast cells: CD13, CD34, CD117, CD33, CD4, CD38, HLA-DR. Karyotype: 46,XY, del(11)(q14~22)[5]/46, idem, del(7)(q21). An immunohistochemical study revealed that granulocytes express MPO and CD15, megakaryocytes express CD31, and myeloblasts express CD117. Mutation V617F of the *JAK2* gene and deletion of three nucleotides in the ninth exon of the *CALR* gene were detected in the DNA of peripheral blood cells (p.K368del c.1102_1104del). The patient received therapy with hydroxycarbamide, azacitidine (75 mg/m^2^ × 7 days), and three courses of small doses of cytarabine. Taking into account the *JAK2*-positivity, an attempt was made to treat with ruxolitinib (15 mg per day), but no significant effect was obtained. Taking into account grade 4 anemia, grade 4 thrombocytopenia, grade 4 neutropenia, an increase in blastosis (28%) and the lack of alternative antitumor therapy, the patient was assigned palliative status. The patient’s clinical data is presented in Table 4. According to NGS data, the allelic load of the *JAK2* V617F mutation was 57%, and the *CALR* p.K368del mutation was 51%. A molecular genetic study of buccal epithelium and hair follicle samples from this patient showed that, unlike the *JAK2* V617F mutation, the *CALR* p.K368del mutation is germline (Table 5). In the described case, ruxolitinib therapy was ineffective, which might indirectly suggest the importance of the *CALR* p.K368del mutation in the pathogenesis of MPN.

## 4. Discussion

The gold standard for routine determination of insertions/deletions in the *CALR* gene is fragment analysis. Most of the *CALR* tests required to confirm the diagnosis of MPN in the clinic are performed using this method. This method is not always precise enough to reliably distinguish frameshift insertion of 5 bp (Type II mutation) from, say, 3 or 6. It should also be noted that the allelic load of somatic mutations in the *CALR* gene can be close to 50% according to both our and the literature data. In our diagnostic practice, we have encountered cases in which germline polymorphisms (mostly in-frame indels) that may not directly relate to the diagnosis were misinterpreted as somatic mutations, thereby making a correct diagnosis challenging. The main purpose of this work was to assess the expected frequency of such cases in order to increase the alertness of doctors and diagnosticians and thus reduce the frequency of possible errors.

There are not enough data on non-type I/II mutations of the *CALR* gene and their significance for the development of MPN in the literature. A 9 bp deletion in the *CALR* gene in a patient with MPN has been reported, although its non-type I/II nature was not confirmed [9]. It can be assumed that non-type I/II mutations of the *CALR* gene can influence the acquisition of somatic mutations and/or interact with classical MPN driver mutations. In the study of Szuber N et al., certain *CALR* gene mutations have been confirmed as non-type I/II in a heterogeneous population of individuals with and without MPN. The authors concluded that germline *CARL* gene variants are not a diagnostic feature of MPN, unlike somatic ones, which are well described in the literature. Predisposition to MPN, related to germline *CALR* gene variants, can also not be concluded [15].

At the National Medical Research Center for Hematology, we have a long history of molecular genetic testing for MPNs. We have accumulated over 8417 records of *CALR* gene mutation tests. This amount of data allowed us to register rare atypical events. Non-type I/II frameshift or nonframeshift mutations were observed in 68 cases. We have found combined mutations in 8 patients (6 patients with *CALR* + *JAK2* V617F mutations and 2 patients with *CALR* + *BCR::ABL* p210 mutations). No second MPN-associated mutation was detected in one patient. It should be noted that the diagnosis of MDS was established in this case. It can be assumed that a germline *CALR* variant without a frameshift may not have pathogenic significance. In other words, only if additional so-called “driver” mutations are present, the development of myeloproliferative neoplasms might be expected.

## 5. Conclusions

Thus, non-type I/II mutations in the *CALR* gene are rare and differ from the classical somatic mutations that determine MPN. The detection of *CALR* mutations of non-I or II types, with VAF close to 50% in MPN, is the basis for suspecting the germline variant and conducting a confirmatory test on a non-hematopoietic tissue sample. The potential role of germline *CALR* variants in the etiology of MPN requires further investigation. The detection of germline *CALR* gene variants should be interpreted with caution to avoid false-positive MPN diagnoses. Perhaps, cases with a non-type I/II *CALR* mutation of a VAF close to 50% found should be suggested for the search for other MPN molecular markers.

## Figures and Tables

**Table 1:** Demographic parameters of patients suspected of MPN (N = 8417).

Age at Onset, Years, (Median)	Sex (Male/Female)	Number of Patients	*CALR* Mutation Status
51	3558/4227	7785	*CALR*−
52	272/360	632	*CALR+*
n/a *	n/a	366	Control group

* not available.

**Table 2:** Clinical parameters of patients with non-type I/II mutations in the *CALR* gene.

Pt #	Age (Years)	Gender	WBC (10^9^/L)	Hb (g/L)	PLT (10^9^/L)	VAF (CALR), %	Additional Mutations	VAF, %	Diagnosis (According to WHO 2022)	Germline
1	72	F	5.28	61	460	50	None	None	MDS	Yes
2	58	M	6.35	107	120	45	*BCR::ABL* p210	0.55	CML + PMF	N/A
3	58	M	7.91	149	454	52	*JAK2* V617F	12.7	MPN	N/A
4	85	F	3.46	91	661	52	*JAK2* V617F	1	ET	N/A
5	91	M	4.04	79	392	52	*JAK2* V617F	100	MPN	Yes
6	77	M	262	66	44	51	*JAK2* V617F	57	PMF	Yes
7	85	M	12.29	127	471	45	*JAK2* V617F	1	ET	N/A
8	62	M	2.47	92	57	50	*JAK2* V617F, *ASXL1* (38%), *U2AF1* (49%), *TET2* (89%)	2	CMML	N/A
9	58	M	1.45	88	41	50	*BCR::ABL* 210	71.2	CML + MPN	Yes

# patient number; M: male; F: female.

**Table 3:** Exact description of non-type I/II *CALR* mutations identified.

Pt #	*CALR* Genetic Variant	COSMIC ID	VAF, %	Deletion
1	*CALR*:NM_004343:exon9:c.1120_1122del:p.K375del	COSV57135468	44	inframe
2	*CALR*:NM_004343:exon9:c.1102_1135del:p.K368Rfs*51	COSV57117058	30	frameshift
3	*CALR*:NM_004343:exon9:c.1132_1134del:p.E381del	None	45	inframe
4	*CALR*:NM_004343:exon9:c.1146_1154delinsTTGTC:p.E383Cfs*46	None	33	frameshift
5	*CALR*:NM_004343:exon9:c.1132_1134del:p.E381del	None	46	inframe
6	*CALR*:NM_004343:exon9:c.1102_1104del:p.K368del	COSV57128367	51	inframe
7	*CALR*:NM_004343:exon9:c.1120_1132del:p.K374Rfs*52	None	39	frameshift
8	*CALR*:NM_004343:exon9:c.1132_1134del:p.E381del	None	46	frameshift
9	*CALR*:NM_004343:exon9:c.1213_1215del:p.E407del	None	44	inframe

# patient number.

**Table 4:** Clinical laboratory data of patient No. 6.

	Result	Reference Value
Hb (g/L)	66	130–160
RBC (1012/L)	2.2	4.50–5.90
PLT (10^9^/L)	44	180.0–320.0
WBC (10^9^/L)	262	4.23–9.00
Blast cells in peripheral blood (%)	28	0.0–0.0
Myelocytes (%)	6	0.0–0.0
Metamyelocytes (%)	2	0.0–0.0
LYM (%)	7	19.0–37.0

**Table 5:** Allelic load of *JAK2* V617F and CALR p.K368del mutations in patient No. 6.

Mutation	Blood	Bone Marrow	Buccal Epithelium
*JAK2* V617F	57%	57%	None
*CALR* p.K368del	51%	55%	55%

## Data Availability

All the data provided within the article.

## Abbreviations

The following abbreviations are used in this manuscript:

*CALR*	**C**alreticulin gene
MPNs	Myeloproliferative neoplasms
CML	Chronic myeloid leukemia
ET	Essential thrombocythemia
PMF	Primary myelofibrosis
VAF	Variant allele frequency
*JAK 2*	Janus kinase 2 gene
WHO	World Health Organization
*MPL*	Myeloproliferative leukemia virus oncogene
*BCR::ABL1*	BCR (Breakpoint Cluster Region) + ABL (Abelson murine leukemia viral oncogene homolog)
DNA	Deoxyribonucleic acid
PCR	Polymerase chain reactions
COSMIC	Catalog of Somatic Mutations in Cancer
